# A Generic Nomogram Predicting the Stage of Liver Fibrosis Based on Serum Biochemical Indicators Among Chronic Hepatitis B Patients

**DOI:** 10.3389/fmed.2021.669800

**Published:** 2021-09-20

**Authors:** Xueying Xu, Wusheng Wang, Qimeng Zhang, Weijie Cai, Mingcheng Wu, Tiantian Qin, Hongbo Liu

**Affiliations:** Department of Health Statistics, School of Public Health, China Medical University, Shenyang, China

**Keywords:** decision tree, nomogram, hepatitis B virus, liver fibrosis, prediction

## Abstract

**Introduction:** Liver fibrosis staging is of great importance for reducing unnecessary injuries and prompting treatment in chronic viral hepatitis B patients. Liver biopsy is not suitable to act a screening method although it is a gold standard because of various shortcomings. This study aimed to establish a predictive nomogram as a convenient tool to effectively identify potential patients with different stages of liver fibrosis for patients with chronic hepatitis B.

**Methods:** A nomogram for multinomial model was developed in a training set to calculate the probability for each stage of fibrosis and tested in a validation set. Fibrosis stages were subgrouped as followed: severe fibrosis/cirrhosis (F3–F4), moderate fibrosis (F2), and nil-mild fibrosis (F0–F1). The indicators were demographic characteristics and biochemical indicators of patients. Continuous indicators were divided into several groups according to the optimal candidate value generated by the decision tree.

**Results:** This study recruited 964 HBV patients undergoing percutaneous liver biopsy. The multinomial model with 10 indicators was transformed into the final nomogram. The calibration plot showed a good agreement between nomogram-predicted and observed probability of different fibrosis stages. Areas under the receiver operating characteristics (AUROCs) for severe fibrosis/cirrhosis were 0.809 for training set and 0.879 for validation set. For moderate fibrosis, the AUROCs were 0.75 and 0.781. For nil-mild fibrosis, the AUROCs were 0.792 and 0.843. All the results above showed great predictive performance in predicting the stage of fibrosis by our nomogram.

**Conclusion:** Our model demonstrated good discrimination and extensibility in internal and external validation. The proposed nomogram in this study resulted in great reliability and it can be widely used as a convenient and efficient way.

## Introduction

Chronic hepatitis B virus (HBV) infection is a major global health problem and affects approximately 360 million persons in the world (1). Liver fibrosis is a critical indicator of anti-virus treatment for patients with HBV infection. A precise assessment of the degree of liver fibrosis is of great importance for guiding clinical treatment and predicting prognosis (2). Liver biopsy has traditionally been considered as a reference standard for assessing and staging fibrosis. But there are several shortcomings such as invasiveness, low compliance, high side-effect, sampling error during the assessment of liver fibrosis (3–6). As a result it is difficult for chronic hepatitis B (CHB) patients to early diagnose or rapid screen liver fibrosis. The non-invasive biomarkers and models have been built to decrease the use of unnecessary liver biopsy. Nowadays, some combined indicators such as index of the relationship of aspartate transaminase to platelete (APRI), fibrosis index based on the four factors (FIB-4), and complex models have been used to predict liver fibrosis as non-invasive methods (7, 8). Although these methods have good diagnostic accuracy, it is pretty hard to get these biomarkers in general hospitals, which always be neglected by researchers. For example, serum microRNA profiles serve as novel biomarkers in a model built by Li et al. (9). Therefore, it is very important to construct the predictive model of liver fibrosis using conventional biomarkers.

In most studies, continuous indicators are directly used to construct predictors or models (5, 10–12). But, as we know, small changes in continuous data have little effect on the prediction and classification. The predictors or models based on the continuous values could will be inefficient in classification or discrimination. The reasonable and effective transformation of the continuous indicator is more beneficial to improve prediction accuracy. For example, the risk of disease changes less with each year of age in a cohort study, and it may be not significant. But when the age increases by 5 years, the risk becomes apparent. Therefore, continuous data were often transformed into ordinal or discrete data in medical and epidemiological research according to the mean, median, percentiles, or reputed clinical threshold (13–16). However, the real impact and characteristics of indicators were not accounted on this condition. Decision trees are simple and effective classification algorithms, which provide human-readable rules of classification (17). In this study, continuous indicators were transformed into ordinal predictors according to the optimal candidate value which was produced by the decision tree. Additionally, a more detailed classification in liver fibrosis is the crucial factor to determine whether to suffer a biopsy. And it is a necessary part for constructing a more reasonable and effective prediction model, which can be more suitable for clinical decision (18).

In order to improve the visualization of results and facilitate the extension of applications, a nomogram is used to build and present predictive models. It can conclude statistical predictive models into a single numerical estimate of the probability of a special event, such as death or recurrence, which is tailored to the profile of an individual patient. Currently, nomograms have been developed rapidly in many fields (19–21). In this study, we aimed to construct a multi-logistic prediction model using routine indicators which could be reasonably grouped by the decision tree, then an intuitive nomogram was determined to clearly and concisely predict the severity of liver fibrosis in CHB patients. It is helpful for clinicians to take reasonable treatment and decision according to the actual situation of patients.

## Materials and Methods

### Study Population

**Figure 1:**
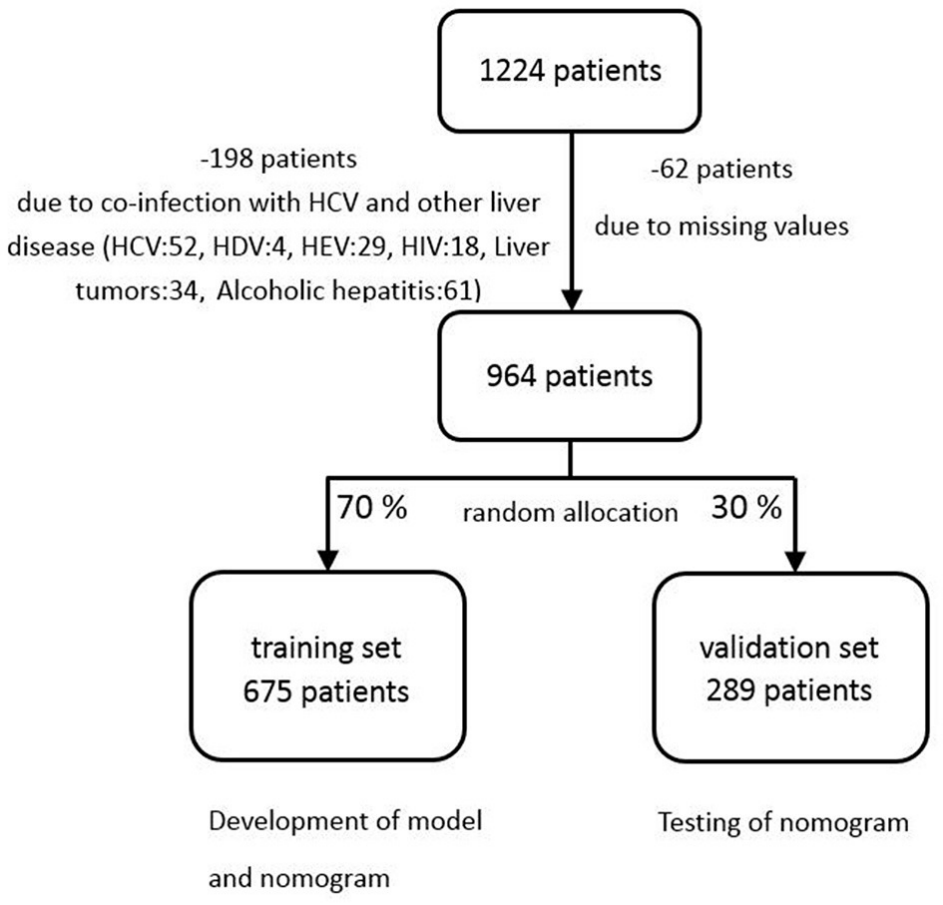
Flow chart of subjects selection.

This study was conducted in 2017 in the Shengjing Hospital of China Medical University. We collected the data of 1,224 patients according to the records in the histology laboratory database. The enrolled subjects were selected according to the following criteria: (1) Hepatitis B surface antigen (HBsAg) was positive at least 6 months, and virus was carried more than 2 years; (2) No co-infection with human immunodeficiency virus (HIV), the hepatitis C or hepatitis D and other liver diseases including chronic ethanol consumption, liver tumors and hepatocellular carcinoma; (3) Before liver biopsy, there is no antiviral therapy in patients; (4) No liver transplantation; (5) Within a week of liver function tests, percutaneous liver biopsy, and serum markers; (6) patients' age ≥ 18. The exclusion criteria were: (1) insufficient liver tissue for the staging of fibrosis; (2) insufficient data on complete blood count or serum markers; (3) There were no serum markers before treatments. If more than one set of laboratory results were available, the results closest to the time of biopsy were used. Among the 1,224 patients collected in the present data, 964 patients were recruited in the final analysis. Two hundred sixty patients were exclude because of incomplete data, co-infection with hepatitis C and other liver disease (Figure 1).

### Patient and Public Involvement

All procedures performed in studies involving patients were in accordance with the ethical standards of the institutional and/or national research committee and with the 1964 Helsinki declaration and its later amendments or comparable ethical standards. Liver biopsy, as an invasive test, was used mainly based on the patient's clinical symptoms, and the patient must sign a consent form. Privacy implications were not involved, and the patients agreed to participate in the study. The study protocol was in accordance with the ethical standards and was approved by the Ethics Committee of China Medical University (CMU6206-1004).

Recruitment of participant into the study was done by health workers based on the inclusion criteria. District and regional health service workers and managers also supported it.

### Laboratory Tests and Clinical Characteristics

All patients were evaluated on standard laboratory parameters. The complete blood count was measured on Hematology Analyzer (Beckman Coulter 5 diff, Miami FL) and clinical chemistry tests were performed using 7150 Analyzer (Hitachi, Japan). All recorded indicators were from blood routine examination, coagulation function, liver and kidney function, serum lipid, myocardial enzyme and demographic characteristics. Thirty-nine variables were excluded in this study, because of literature and medical background (28 predictors such as Chlorine, Urea, Uric acid), as well as over-missing values (11 predictors such as C-reactive protein, Hepatitis b E antigen, HBeAb, HBV-DNA).

The variable “sign” is primarily considered as an indicator of clinical feature, which represents the status and symptoms of patients. If a patient had both liver palms and spider nevus, the “sign” was assigned to 2. If a patient had either liver palms or spider nevus, the “sign” was assigned to 1. If a patient has neither liver palms nor spider nevus, the “sign” was assigned to 0.

### Liver Histological Examination

Patients received percutaneous liver biopsy with automatic fare cut biopsy needle after signing the informed consent. All the samples were at least 10 mm in length and 1 mm in width. Two pathologists who had no clinical information of patients evaluated all biopsy specimens. The level of fibrosis was evaluated semi-quantitatively according to the METAVIR scoring system, which had previously been applied in other reports on CHB (22). Fibrosis was classified from F0 to F4 stages: F0 for no fibrosis, F1 for portal fibrosis without septa, F2 for few septa, F3 for numerous septa without cirrhosis and F4 for cirrhosis.

### Statistical Analysis

The recruited patients were randomly divided into two sets, training set and validation set, by a ratio of 7:3. The training set was used to generate a plausible model, and the validation set was used to accomplish the validation and assess the performance of the model (Figure 1). Categorical variables were demonstrated with percentage, and were compared with the chi-squared test. Quantitative variables were shown as median with interquartile range (IQR), which were compared with Mann-Whitney tests. All *P*-values reported were 2-sided, and *P* < 0.05 was considered to be statistical significance. The analysis was carried out by SAS 9.4 and R.3.6.0 software (http://www.R-project.org).

### Decision Tree

All recruited patients were included in a decision tree, and the result of individual biopsy was used as the classification of the decision tree. Then the optimal threshold value was calculated for every single covariate. Based on the analysis of the decision tree, all of the predictors are divided into two or more sections. This analysis was carried out using SPSS 20.

### Nomogram

Nomogram is a graphic calculating tool helping clinicians quickly evaluate patients with specific models in a visual way, which does not require complex interpretation by computer software. It is based on multivariate regression analysis that integrates multiple indicators and then uses segments with scales to plot on the same plane at a certain scale to express the interrelations between variables in the prediction model.

A multinomial model was developed using categorized predictors and biopsy information. The classification of fibrosis stages (response variable) was divided into three categories: nil-mild fibrosis (F0–F1), moderate fibrosis (F2), and severe fibrosis/cirrhosis (F3–F4). The independent predictors included in the model were basic information and biochemical indicators. When carrying out a multinomial regression model, stepwise forward selection procedures were used to select the predictors in the model.

The established model was translated into a nomogram to display its outcome and corresponding probabilities conveniently. We can get the total point of every patient by accumulating points for each line. Then it is easy to get the corresponding lp (linear predicator) and the exponentiated point by drawing a vertical line from the total point axis straight to Exp(lp.m) or Exp(lp.s) axis, and then calculate the final probabilities of three fibrosis stages through the following formulas:


(1) $$\begin{array}{l} P_{F0-F1}=\frac{1}{1+Exp\left(lp\cdot m\right)+Exp\left(lp\cdot s\right)} \end{array}$$

(2) $$\begin{array}{l} P_{F2}=\frac{Exp\left(lp\cdot m\right)}{1+Exp\left(lp\cdot m\right)+Exp\left(lp\cdot s\right)} \end{array}$$

(3) $$\begin{array}{l} P_{F3-F4}=\frac{Exp\left(lp\cdot s\right)}{1+Exp\left(lp\cdot m\right)+Exp\left(lp\cdot s\right)} \end{array}$$

Of course, we can also calculate Exp(lp) without finding it in the plot. The Exp(lp) equal to e^*ip*^, and lp is the linear predictor that you can get from nomogram.

### Model Evaluation

To get bias-corrected estimates of predicted vs. observed values based on non-parametric smoothers, we established calibration plots using bootstrapping. The receiver operating characteristic curves (ROC) were constructed to analyze the accuracy of the model. Diagnostic accuracy for discriminating the stage of fibrosis was expressed as the area under the receiver operating characteristic curve (AUROC) for each outcome probability, both in the training set and validation set. We can also get the sensitivity, specificity and likelihood ratio from it.

## Results

### Population Characteristics

The basic characteristics of the 964 study patients are shown in Table 1. According to the METAVIR score, 529 (54.88%) patients are in F0 stage, 213 (22.10%) patients are in F1 stage, 145 (15.04%) patients are in F2 stage, 74 (7.68%) in F3 stage and 3 (0.31%) patients are in F4 stage. Three continuous variables, Total bilirubin (TBIL), Hydroxybutyrate dehydrogenase (HBDH), D-Dimer, and one binary variable with no statistical significance were excluded from the next step (*P* > 0.05) and the rest of variables all showed statistical significance within different levels of liver fibrosis (*P* < 0.05). And there is no difference between training set and validation set (Supplementary Table 1).

**Table 1:** Clinical and laboratory characteristics of HBV patients in different levels.

**Variables**		**F0/F1 (*n* = 742)**	**F2 (*n* = 145)**	**F3/F4 (*n* = 77)**	***P*-value**
Gender	Male	487 (65.63)	101 (69.66)	54 (70.13)	0.5088
[n (%)]	Female	255 (34.37)	44 (30.34)	23 (29.87)	
Smoking	Yes	135 (18.19)	35 (24.14)	24 (31.17)	0.0110
[n (%)]	No	607 (81.81)	110 (75.86)	53 (68.83)	
Drinking	Yes	148 (19.95)	38 (26.21)	23 (29.87)	0.0472
[n (%)]	No	594 (80.05)	107 (73.79)	54 (70.13)	
SIGN	0	598 (80.59)	119 (82.07)	48 (62.34)	0.0002
[n (%)]	1	121 (16.31)	16 (11.03)	22 (28.57)	
	2	23 (3.10)	10 (6.90)	7 (9.09)	
Age (years)		34 (26–41)	36 (27–43)	38 (32–44)	0.0026
A/G		1.56 (1.4–1.7)	1.5 (1.3–1.6)	1.42 (1.25–1.6)	<0.001
ALT (UI/ml)		45 (26–78.55)	65 (34–119)	69 (38–106)	<0.001
AST (UI/ml)		29 (22–47)	45 (28–78)	42 (29–86)	<0.001
ALB (g/L)		42.6 (40.6–45.2)	41.9 (39.7–43.4)	42 (39.1–43)	<0.001
ALP (U/L)		73 (60–84.7)	79.55 (63.8–103)	79.55 (70.5–110)	0.0007
APOB (g/L)		0.85 (0.71–1)	0.77 (0.65–0.92)	0.78 (0.62–0.99)	0.0123
DBIL (μmol/L)		4.2 (3.1–5.2)	4.73 (3.6–6)	4.73 (3.8–6.6)	<0.001
TBIL (μmol/L)		1 1.9 (9.2–14.6)	13.19 (9.5–15.9)	13.19 (10.7–17.6)	0.0553
CHE (U/L)		7927.91 (6,732–9,363)	7,173 (5,877–8,294)	7,137 (5,746–7,927.91)	<0.001
CYSC (mg/L)		0.82 (0.72–0.92)	0.84 (0.71–0.97)	0.9 (0.77–1.03)	0.0312
CHOL (mmol/L)		4.31 (3.85–4.88)	4.11 (3.68–4.72)	4.04 (3.65–4.76)	0.0069
GGT (U/L)		27 (17–44.91)	44 (25–72)	44.91 (29–86)	<0.001
GLU (mmol/L)		5.17 (4.87–5.45)	5.16 (4.79–5.52)	5.13 (4.86–5.7)	0.0331
HBDH (U/L)		141.3 (126.5–156)	144.6 (127.3–160)	145 (130–162)	0.4098
TBA (μmol/L)		4.8 (2.6–8.95)	8.5 (4.45–12.1)	8.8 (4.4–12.1)	0.0101
AFP (μg/L)		2.63 (1.87–4.3)	4.01 (2.6–7.4)	5.11 (3–9.31)	<0.001
APTT (s)		30.55 (28–33)	32 (29–35)	32 (28–36)	<0.001
D-Dimer (μg/L)		97 (59–156)	88 (50–145)	100 (65–160)	0.0589
FIB (g/L)		2.4 (2.1–2.8)	2.3 (2–2.6)	2.4 (2.1–2.6)	0.007
PT (s)		11.3 (10.8–11.9)	11.6 (11.1–12.2)	11.8 (11.2–12.5)	<0.001
TT (s)		15.9 (15.4–17.4)	16.5 (15.9–18.3)	16.5 (15.8–18.6)	<0.001
MPV (fl)		9.2 (8.1–10.24)	9.8 (8.7–11)	9.2 (8.4–10.1)	0.0012
PDW (fl)		16.1 (14.6–16.59)	15.24 (13.4–16.5)	16.3 (15.24–16.7)	0.0109
PLT (10^9^/L)		183.5 (153.1–220)	150 (127–184)	149 (119–180)	<0.001

*Data are presented as number (%) or median (interquartile range). A/G, Albumin/globulin; ALT, Alanine aminotransferase; AST, Aspartate aminotransferase; ALB, Albumin; ALP, Alkaline phosphatase; APOB, Apolipoprotein-B; DBIL, Direct bilirubin; TBIL, Total bilirubin; CHE, Cholinesterase; CYSC, CystatinC; CHOL, Cholesterol; GGT, γ-glutamyl transpeptidase; GLU, Glucose; HBDH, Hydroxybutyrate dehydrogenase; TBA, Total bile acid; AFP, Alpha fetoprotein; APTT, Activated partial thromboplastin time; FIB, Plasma fibrinogen; PT, Prothrombin time; TT, Thrombin time; MPV, Mean platelet volume; PDW, platelet distribution width; PLT, Platelets count*.

### Transformation of Indicators

In this study, continuous indicators were transformed into discrete ones according to the optimal candidate value produced by the analysis of the decision tree. Nine indicators were transformed into dichotomous indicators. Eight indicators were transformed into three-category indicators. Three indicators were transformed into four-category indicators and two indicators were transformed into five-category indicators. The specific classification and optimal candidate values of final indicators were shown in Table 2.

**Table 2:** The levels and optimal candidate values of final indicators.

**Factors**	**Score (actual range)**				
	**0**	**1**	**2**	**3**	**4**
AGE	≤ 31	>31			
CHOL	≤ 4.09	>4.09			
APTT	≤ 35.7	>35.7			
PT	≤ 11.2	>11.2			
PDW	≤ 15.2	>15.2			
TT	≤ 15.3	15.3–16.4	>16.4		
ALP	≤ 51.3	51.3–109	>109		
GGT	≤ 24	24–54	54–85	>85	
PLT	≤ 137	137–166	166–223	>223	
AFP	≤ 1.47	1.47–2.87	2.87–3.45	3.45–6.78	>6.78

*Every original indicator of patients can be transformed into score (0, 1, 2, 3, or 4) according to this table, and then the scores can be used in the nomogram plot to calculate the risk of liver fibrosis*.

### Multinomial Logistic Regression

Based on multinomial logistic regression, we constructed predictive models of the degree of liver fibrosis in the training set. Ten biochemical markers were included in the final model with nil-mild fibrosis as a reference. Table 3 showed relative factors of liver fibrosis. They are age (AGE), Alkaline phosphatase (ALP), Cholesterol (CHOL), γ-glutamyl transpeptidase (GGT), Alpha fetoprotein (AFP), Activated partial thromboplastin time (APTT), Prothrombin time (PT), Thrombin time (TT), platelet distribution width (PDW), and Platelets count (PLT).

**Table 3:** Multinomial estimates from the final multinomial logistic regression model.

**Predictive determinants**	**Moderate fibrosis vs. Nil-mild fibrosis**			**Severe fibrosis/cirrhosis vs. Nil-mild fibrosis**		
	**β**	**OR (95%CI)**	***P-value***	**β**	**OR (95%CI)**	***P-value***
AGE	0.203	1.224 (0.752–1.994)	0.416	0.994	2.702 (1.343–5.438)	0.005
ALP	0.128	1.137 (0.653–1.979)	0.651	1.075	2.929 (1.449–5.92)	0.003
CHOL	−0.521	0.594 (0.369–0.956)	0.032	−0.603	0.547 (0.296–1.01)	0.054
GGT	0.323	1.382 (1.069–1.787)	0.014	0.296	1.345 (0.964–1.876)	0.081
AFP	0.391	1.478 (1.195–1.829)	<0.001	0.299	1.348 (1.021–1.78)	0.035
APTT	0.608	1.838 (0.936–3.607)	0.077	1.079	2.941 (1.341–6.451)	0.007
PT	0.531	1.701 (1.028–2.815)	0.039	0.675	1.964 (0.999–3.862)	0.05
TT	0.629	1.875 (1.316–2.673)	0.001	0.501	1.65 (1.052–2.588)	0.029
PDW	−0.522	0.594 (0.352–1.001)	0.051	0.543	1.721 (0.807–3.674)	0.16
PLT	−0.39	0.677 (0.536–0.854)	0.001	−0.578	0.561 (0.411–0.766)	<0.001

*ALP, Alkaline phosphatase; CHOL, Cholesterol; GGT, γ-glutamyl transpeptidase; AFP, Alpha fetoprotein; APTT, Activated partial thromboplastin time; PT, Prothrombin time; TT, Thrombin time; PDW, platelet distribution width; PLT, Platelets count*.

### Multinomial Nomogram

**Figure 2:**
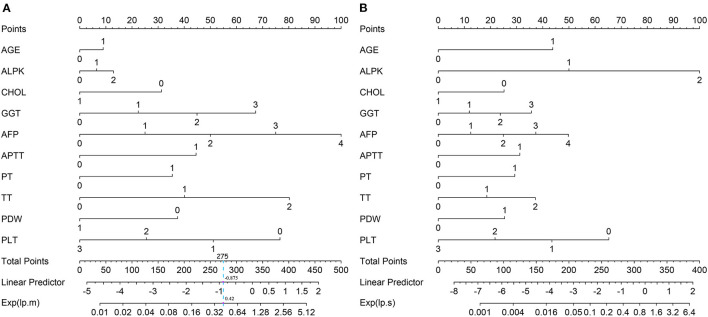
The multinomial nomogram for the prediction of mild-moderate fibrosis **(A)** and severe fibrosis/cirrhosis **(B)**.

The nomogram enabled to calculate the probabilities of moderate (Figure 2A) and severe fibrosis/cirrhosis (Figure 2B). We can get the total point of every patient by accumulating points for each line, and the corresponding linear predictor (lp). We can also get the Exp(lp) by drawing a vertical line from the linear predictor axis straight to Exp(lp) axis, and then calculate the final probabilities of three fibrosis stages through the above mentioned formulas.

### Calibration Plot

**Figure 3:**
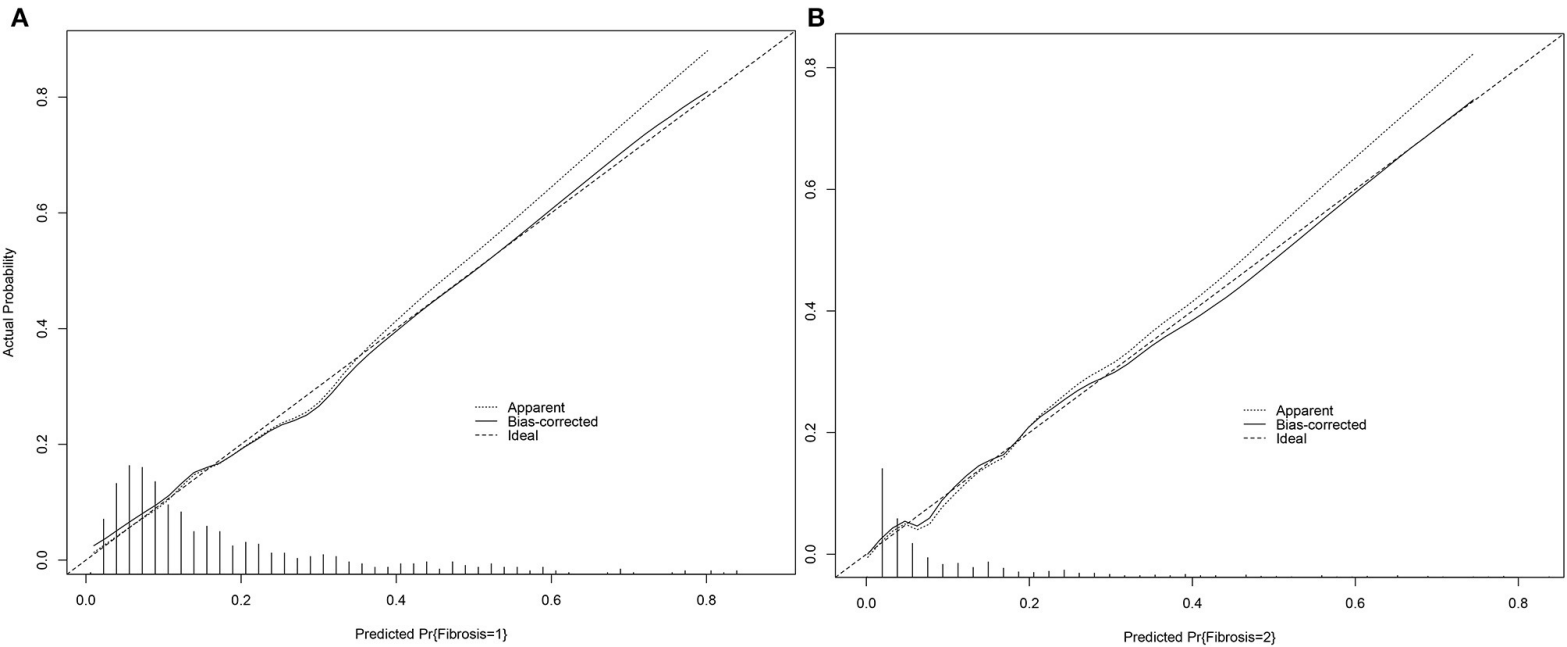
Calibration plot of nomogram for mild-moderate fibrosis **(A)** and severe fibrosis/cirrhosis **(B)**.

The calibration plot only tells us the bias of a classifier and has no connection with the classification quality. The dashed line indicates the ideal model in which predicted and actual probabilities were perfectly identical. The dotted line indicates actual model performance. The solid line presents the bootstrap corrected performance of our model. The bootstrap calibration plot (Figure 3A) indicated a good agreement between nomogram-predicted and observed probability of different fibrosis level for mild-moderate fibrosis group. However, it showed a good agreement for severe fibrosis group (Figure 3B). But the track of dotted line and solid line is different with ideal line which indicated predictions may slightly differ from reality.

### Model Validation

**Figure 4:**
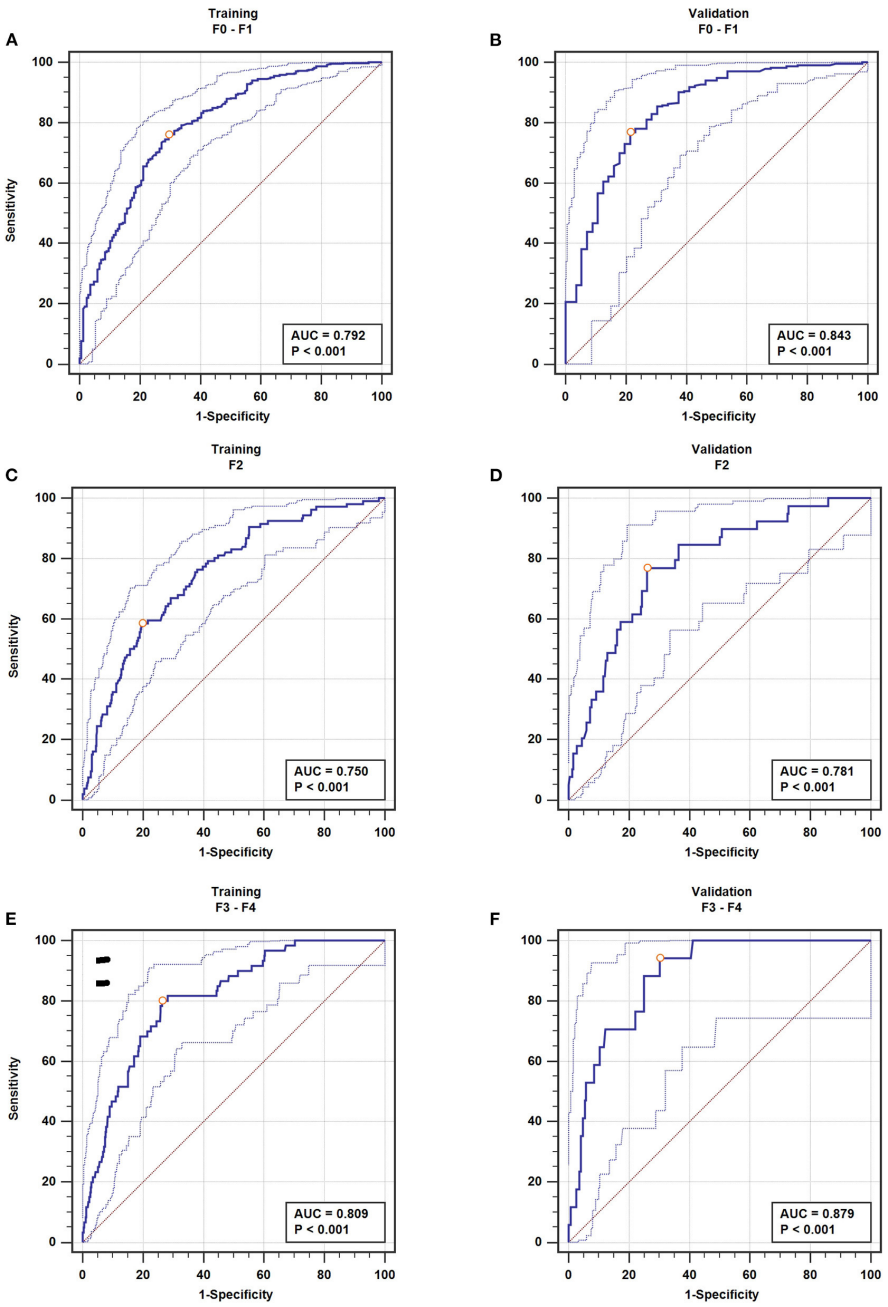
The AUROC of fibrosis. nil-mild fibrosis **(A)**, moderate fibrosis **(C)**, severe fibrosis/cirrhosis **(E)** in the training set; nil-mild fibrosis **(B)**, moderate fibrosis **(D)**, severe fibrosis/cirrhosis **(F)** in the validation set.

For nil-mild fibrosis, we got AUROCs of 0.792 (95% CI 0.760–0.822) for the training set (Figure 4A) and 0.843 (95% CI 0.796–0.883) for the validation set (Figure 4B). For moderate fibrosis, our model enabled correct identification of patients with AUROCs of 0.750 (95% CI 0.715–0.782) for the training set (Figure 4C) and 0.781 (95% CI 0.729–0.827) for the validation set (Figure 4D). For severe fibrosis/cirrhosis (F3–F4), the model showed a good discrimination performance with AUROCs of 0.809 (95% CI 0.778–0.838) in the training set (Figure 4E) and 0.879 (95% CI 0.836–0.915) maintained in the validation set (Figure 4F), which demonstrated an intrinsic robust performance of the predictive model in terms of discrimination.

The detail information of the model in predicting fibrosis was shown in Table 4. The model predicted severe fibrosis with a sensitivity of 80.00% and a specificity of 73.66% in the training set at the optimal cutoff. In the validation set, the same cutoff yielded a sensitivity of 94.12% and a specificity of 69.85% accompanied with an LR+ 3.12 and LR− 0.084. Choosing the point on the ROC curve corresponding to the best cutoff, the model predicted moderate fibrosis with a sensitivity of 58.49% and a specificity of 80.14% in the training set with an LR+ 2.95, and LR− 0.52. In the validation set, the same cutoff yielded a sensitivity of 76.92% and a specificity of 74.00% accompanied with an LR+ 2.96 and LR− 0.31. In the same way, the model predicted nil-mild fibrosis with a sensitivity of 76.03% and a specificity of 70.48% accompanied with an LR+ 2.58 and LR− 0.34. In the validation set, the same cutoff yielded a sensitivity of 76.83% and a specificity of 78.57% accompanied with an LR+ 3.59 and LR− 0.29.

**Table 4:** The detail information of the multinoimal nomogram in predicting of fibrosis.

**Fibrosis**	**Nil-mild fibrosis**		**Moderate fibrosis**		**Severe fibrosis/cirrhosis**	
**Data set**	**Training**	**Validation**	**Training**	**Validation**	**Training**	**Validation**
Cut-off	0.7302		0.2135		0.0967	
Sensitivity (%)	76.03 (72.1–79.7)	76.83 (70.9–82.1)	58.49 (48.5–68.0)	76.92 (60.7–88.9)	80.0 (67.7–89.2)	94.12 (71.3–99.9)
Specificity (%)	70.48 (62.9–77.3)	78.57 (65.6–88.4)	80.14 (76.7–83.3)	74.00 (68.1–79.3)	73.66 (70.0–77.1)	69.85 (64.0–75.2)
LR+	2.58 (2.0–3.3)	3.59 (2.2–5.9)	2.95 (2.3–3.7)	2.96 (2.3–3.9)	3.04 (2.5–3.6)	3.12 (2.5–3.9)
LR–	0.34 (0.3–0.4)	0.29 (0.2–0.4)	0.52 (0.4–0.7)	0.31 (0.3–0.6)	0.27 (0.2–0.5)	0.084 (0.01–0.6)
AUC	0.792 (0.760–0.822)	0.843 (0.796–0.883)	0.750 (0.715–0.782)	0.781 (0.729–0.827)	0.809 (0.778–0.838)	0.879 (0.836–0.915)

*LR+, positive likelihood ratio; LR−, negative likelihood ratio; AUC, Area under of ROC curve*.

## Discussion

Liver fibrosis is known as the major problem causing morbidity and mortality in chronic HBV patients. The evaluation of liver fibrosis stage in CHB patients is not only conducive to precision treatment by doctors, but also can reduce the burden of patients (23). We investigated HBV patients who had liver biopsies in the same hospital, and over 50% of them were actually in F0 stage. However, they are also at risk from unnecessary biopsies. Therefore, it is necessary to find a non-invasive method to determine whether a patient must further undergo an invasive procedure. Several biomarkers and combining markers are related to liver fibrosis and many non-invasive models have been suggested as good choices for screening liver fibrosis in order to overcome the limitations of liver biopsy (24–27). In our study, routine biomarkers and clinical markers were used to establish noninvasive predictive models for liver fibrosis. The final model included routine biomarkers which can be easily obtained from general hospital and even in local clinics with laboratory, such as AGE, ALPK, CHOL, GGT, AFP, APTT, PT, TT, PDW, PLT, which is conducive to the expansion of clinical applications.

Decision tree classification with a single classifier has been very successful in general classification problems. It provides human-readable rules of classification (28). The optimal separating points and the number of categories are based on the characteristics of every indicator and its influence on the target outcome, and the relationship between the outcome and indicators make each classification more reasonable. But, in several researches, continuous indicators were directly used without considering the fact that the tiny changes in a primitive continuous variable may obscure its role in the final model, which may result in this significant indicator being excluded from the model (29–31). On the other hand, the impact of extreme values could be reduced by transforming variables into categorical variables before the modeling process, although some of the original information may be lost. Classification of continuous variables by decision trees has been applied and the good result had been obtained (28). We used the decision tree to automatically classify 22 meaningful continuous indicators into dichotomous indicators, three-category indicators, four-category indicators or five-category indicators. The classification can better reflect the influence of different levels of indicators on liver fibrosis.

In our study, a multinomial logistic regression was conducted to build a predictive model instead of an ordinal logistic regression in view of the limitations of the application conditions of ordinal logistic regression. In addition, covariates' effects are the same independently of response categories considered in ordinal logistic regression model, but in practice, we suspect that a set of coefficients does not contribute to good predictive performance. So, the multinomial model became our ultimate choice. We put the multinomial logistic regression formula into an obvious nomogram plot to eliminate the tedious calculations. The nomogram accompanied with the formula can be used to calculate each patient's probability of two kinds of fibrosis in CHB patients. As a method to identify the high-risk or low-risk individual, it is easy and fast, and saves public resources. In our study, the nomogram is very effective in predicting the degree of liver fibrosis in more detail, such as nil-mild fibrosis, moderate fibrosis, and severe fibrosis. These showed good discrimination ability for nil-mild fibrosis with AUROCs of 0.792 in the training set and 0.843 in the validation set. For moderate fibrosis, AUROCs were 0.750 and 0.781. Especially for severe fibrosis, the nomogram showed better accuracy with AUROCs of 0.809 and 0.879. Compared with other validated widely non-invasive models (32), such as FIB-4 with AUROC of 0.766, APRI with AUROC of 0.728, Wang I with AUROC of 0.766, PP with AUROC of 0.772, our model got a better result. Though WHO recommends APRI as the preferred non-invasive test to assess significant fibrosis or cirrhosis and FIB-4 to detect of fibrosis stages ≥F3 (33), the AUROC of our model is bigger than theirs. And even better than Forns Index, another serum non-invasive fibrosis test, has cholesterol more than FIB-4 in the formula (34). Transient elastography performed with FibroScan (Echosens, Paris) has been evaluated widely and has a good performance of predicting cirrhosis, which is corroborated by Guidelines Development Group. But it requires more expensive equipment and professional technicians, so they considered it was the most useful test for the assessment of cirrhosis in middle-income countries. Some researchers also included transient elastography as a variable in logistic regression established the nomogram. And it showed good prediction results (35). Compared with it, the AUROC of our nomogram is close to it, even our regression model has a better predictive effect, but only the routine serum biochemical indicators are used. Our model has more variables than other methods, but these variables are routine blood biochemical indicators, which are easy to implement in general medical examinations. The variables of our model are also available when the variables of the APRI or other model are obtained, so it is not difficult to practice. The final score of each patient accumulated through different variables can be used to estimate the risk of liver fibrosis, which is intuitive and more applicable to the use of primary hospitals. In addition, continuous indicators were transformed into ordinal predictors by the decision tree before multinomial logistic regression in our nomogram. It could improve prediction accuracy and made the AUROC bigger than nomogram by traditional regression model (36).

However, there were some limitations in our study. It was conducted in a specialized department for infectious diseases. All enrolled individuals were inpatients, not a completely random sample of all CHB patients. These inpatients could pay more attention to their own health. They are hospitalized as soon as possible to slow down the development of the disease. However, many CHB patients don't care about their health. They have not been hospitalized in time, and their condition has developed into fibrosis without knowing it. Therefore, our study might potentially underestimate the percentage of mild fibrosis in CHB patients. In addition, owing to the limitation of retrospective investigation, we did not collect some information such as HBV genotypes, virus load, dietary habit, use of health food (37). Therefore, we could not determine whether these variables should be included in the model.

In conclusion, this study presents nomograms covers mild-moderate fibrosis, and severe fibrosis, and it can be effectively used to predict the degree of liver fibrosis in CHB patients. We have confirmed that the nomogram based on decision tree could improve the more accuracy of individualized prediction and clinical benefit.

## Data Availability Statement

The raw data supporting the conclusions of this article will be made available by the authors, without undue reservation.

## Ethics Statement

The studies involving human participants were reviewed and approved by the Ethical Committee Group of China Medical University (CMU6206-1004). The patients/participants provided their written informed consent to participate in this study. The patients/participants provided their written informed consent to participate in this study.

## Author Contributions

XX conducted the design of study, performed statistical analysis, and wrote the initial manuscript after consultation with the other authors. HL improved the design, revised the manuscript, and approved the final version. WW collected the preliminary data and helped revise the manuscript. QZ participated in the design and acquisition of preliminary data. WC collected and sorted the preliminary data. MW and TQ participated in the collection of the data. All authors have read and approved the submitted version of the manuscript.

## Funding

This study was partly supported by the Social Sciences Foundation of Liaoning Province (Grant No. L18ATJ001) to HL. None of the funders had any role in the design of the study and collection, analysis, and interpretation of data and in writing the manuscript.

## Conflict of Interest

The authors declare that the research was conducted in the absence of any commercial or financial relationships that could be construed as a potential conflict of interest.

## Publisher's Note

All claims expressed in this article are solely those of the authors and do not necessarily represent those of their affiliated organizations, or those of the publisher, the editors and the reviewers. Any product that may be evaluated in this article, or claim that may be made by its manufacturer, is not guaranteed or endorsed by the publisher.
