# Supplementary material for: A Generic Nomogram Predicting the Stage of Liver Fibrosis Based on Serum Biochemical Indicators Among Chronic Hepatitis B Patients
Source: Front Med (Lausanne). 2021 Sep 20;8:669800. doi: 10.3389/fmed.2021.669800 (PMC8488358; doi:10.3389/fmed.2021.669800)
Supplement: Supplementary file 1 [file Table_1.docx]

Table S1 Clinical and laboratory characteristics of HBV patients in different sets

| variable | Training set  (n=675 ) | | Validation set  (n=289) | | *P* value |
| --- | --- | --- | --- | --- | --- |
| AGE (year) | 35 | (27-42) | 34 | (27-42) | 0.7575 |
| A/G | 1.50 | (1.40-1.70) | 1.54 | (1.39-1.70) | 0.5452 |
| ALT (UI/ml) | 49 | (28-84) | 47 | (28-83) | 0.7555 |
| AST (UI/ml) | 31 | (23-52) | 32 | (22-53) | 0.8097 |
| ALBG (g/L) | 42.56 | (40.30-45.10) | 42.56 | (40.29-44.50) | 0.1990 |
| ALP K(U/L) | 73.0 | (61.0-87.4) | 75.4 | (60.0-87.6) | 0.6410 |
| APOB (g/L) | 0.83 | (0.69-0.97) | 0.86 | (0.68-1.02) | 0.9376 |
| CHE (U/L) | 7828 | (6488-9166) | 7698 | (6371-8958) | 0.1583 |
| CYSC (mg/L) | 0.82 | (0.72-0.94) | 0.84 | (0.73-0.96) | 0.8381 |
| CHOL (mmol/L) | 4.26 | (3.81-4.83) | 4.27 | (3.82-4.85) | 0.7199 |
| GGT (U/L) | 29 | (18-47) | 30 | (18-45) | 0.3698 |
| TBA (μmol/L) | 5.12 | (2.80-9.09) | 6.25 | (3.01-10.72) | 0.1193 |
| AFP (μg/L) | 2.82 | (1.89-5.11) | 2.96 | (2.05-4.92) | 0.2044 |
| APTT (s) | 30 | (28-33) | 31 | (28-33) | 0.5129 |
| D-Dimer (μg/L) | 98 | (57-163) | 97 | (59-149) | 0.2697 |
| FIB (g/L) | 2.4 | (2.1-2.8) | 2.3 | (2.1-2.7) | 0.9930 |
| PT (s) | 11.5 | (10.9-12) | 11.4 | (10.9-12.1) | 0.4496 |
| TT (s) | 16.10 | (15.40-18.00) | 16.10 | (15.50-18.15) | 0.8960 |
| MPV (fl) | 9.0 | (8.2-10.2) | 9.1 | (8.1-10.1) | 0.2695 |
| PLT (10^9^/L) | 178.0 | (142.1-215.0) | 176.5 | (143.0-210.5) | 0.6588 |

Data are presented as median (interquartile range). A/G: Albumin/globulin; ALT:Alanine aminotransferase; AST:Aspartate aminotransferase; ALB:Albumin; ALP:Alkaline phosphatase; APOB: Apolipoprotein-B; CHE:Cholinesterase; CYSC:CystatinC; CHOL:Cholesterol; GGT:γ-glutamyl transpeptidase; TBA:Total bile acid; AFP:Alpha fetoprotein; APTT:Activated partial thromboplastin time; FIB:Plasma fibrinogen; PT:Prothrombin time;TT:Thrombin time; MPV:Mean platelet volume; PLT:Platelets count;
